# Medical Men and Dentistry

**Published:** 1895-05

**Authors:** Geo. W. Warren

**Affiliations:** Philadelphia.


ARTICLE XI.

### MEDICAL MEN AND DENTISTRY.

BY DR. GEO. W. WARREN, PHILADELPHIA.

As further evidence of the need of a higher standard of dental knowledge for medical practitioners, especially those who incidentally figure as teachers, we cite the following: A well-known surgeon, who is also an author and a professor, cautions his students to "be sure, before extracting a tooth, to cut the *ligamentum dentis*." He then relates that its discoverer "first detected it by means of a microscope, in the maxilla of a hog, and afterward demonstrated it in the human subject." Another interesting instance is given by a contributor to one of our journals. He was requested by a practicing physician, who was also an editor of a medical journal, to see a child who was "suffering from some trouble which was difficult to diagnose —a most extraordinary and distressing case. A portion of the superior maxillary bone was denuded; integument sloughing away," etc. On examination, there was found protruding, some distance above the gum margin, the denuded root of a decayed temporary tooth, such as we frequently see; this being the sole cause for the alarm. Wc frequently learn of physicians allowing patients to suffer from alveolar abscess, putting them to bed with the information that they are suffering from "neuralgia." And quite recently a lady called on us with the request to have a small amalgam filling removed. It was the only alloy filling she had, and was in good condition. She had been sent by her physician, who claimed he could not cure a throat affection, from which she was suffering, as long as the filling was retained.

Our literature is replete with authentic reports of such bases, but an excellent illustration of the point in hand, one that has never been published, we take from the practice of Professor Peirce. A well known neurologist had under his care a lad suffering from chorea. The case was presented to Dr. Peirce, who, on examination, found several persistent deciduous teeth, interfering with the normal eruption of the bicuspids, and these at his suggestion were removed, and within a few days the distressing symptoms disappeared. Under the care of the same was a girl ubject to attacks of epilepsy, which were thought by the attending physician to be incurable, but on the removal of several deciduous teeth and two deceased first molars, the marked improvement was evidence conclusive that dental irritation was in this case also an important factor.

A well-known dental writer, the late Professor Richardson, has truly said that it is the reproach of curative medical science that 'patients are daily drugged for the relief of maladies which, being purely symptomatic of, or dependent on, dental diseases or irritation, are diagnosed and obstinately and perseveringly treated as idiopathic affections.

It is also a reproach of conservative medical science that countless numbers of teeth are, year after year, uselessly and mercilessly sacrificed through the direct agony, or complicity, of many medical practitioners; and a reproach of sanitary medical science that, while the greatest circumspection is displayed in guarding the many approaches to the citadel of health, one of the most exposed and vulnerable points, whose defense is essential to the general safety, should be left so entirely uncared for.

Of course, all medical practitioners are not thus perverse and stupid. We number among our clients several of the most active physicians, who are always anxious, through personal and professional intercourse, to secure any information of value. Many of us, too, are called in consultation by physicians who recognize the close relationship of dentistry and medicine. But these are the exception—they are the salt of the profession. It is the average medical practitioner (which is the majority) to whom we allude.—*Independent.*

